# Identification of histone acetylation markers in human fetal brains and increased H4K5ac expression in neural tube defects

**DOI:** 10.1002/mgg3.1002

**Published:** 2019-10-14

**Authors:** Dan Li, Chunlei Wan, Baoling Bai, Haiyan Cao, Changyun Liu, Qin Zhang

**Affiliations:** ^1^ Weifang Medical University Weifang China; ^2^ Beijing Municipal Key Laboratory of Child Development and Nutriomics Capital Institute of Pediatrics Beijing China

**Keywords:** histone acetylation, human fetal brain, mass spectrometry, neural tube defects

## Abstract

**Background:**

Neural tube defects (NTDs) are severe common birth defects that result from a failure in neural tube closure (NTC). Our previous study has shown that decreased histone methylation altered the regulation of genes linked to NTC. However, the effect of alterations in histone acetylation in human fetuses with NTDs, which are another functional posttranslation modification, remains elusive. Thus, we aimed to identify acetylation sites and changes in histone in patients with NTDs.

**Methods:**

First, we identified histone acetylation sites between control human embryonic brain tissue and NTDs using Nano‐HPLC‐MS/MS. Next, we evaluated the level of histone acetylation both groups *via* western blotting (WB). Finally, we used LC‐ESI‐MS and WB to compare whether histone H4 acetylation was different in NTDs.

**Results:**

A total of 43 histone acetylation sites were identified in human embryonic brain tissue, which included 16 novel sites. Furthermore, we found an increased histone acetylation and H4K5ac in tissue with NTDs.

**Conclusion:**

Our result present a comprehensive map of histone H4 modifications in the human fetal brain. Furthermore, we provide experimental evidence supporting a relationship between histone H4K5ac and NTDs. This offers a new insight into the pathological role of histone modifications in human NTDs.

## Graphical abstract

A total of 43 histone acetylation sites were identified in human embryonic brain tissue, which included 16 novel sites. Furthermore, we found increased histone acetylation and H4K5ac in tissue with NTDs.

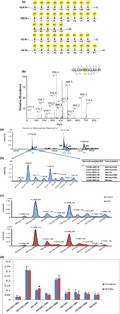

## 1 INTRODUCTION

Neural tube defects (NTDs) form a group of severe congenital malformations, including anencephaly, spina bifida, and encephalocele (Lei, Jingru, Yanjun, Hui, & Aiguo, 2017). The incidence of NTDs is approximately 1 in 1,000, and is estimated reach to 4–10 in 1,000 in northern China (Gu et al., 2007; Put, Straaten, Trijbels, & Blom, 2001). The etiology of NTDs is largely unknown; however, it is considered to be multifactorial, involving multiple interacting genes and environmental factors. The mechanisms by which environmental factors affect the process of neural tube closure and their critical interaction with genetic factors remain unknown.

Previous research has indicated that aberrant histone acetylation may cause NTDs. Shyamasundar et al have found that hyperglycemia alters epigenetic mechanisms, including histone methylation and histone acetylation, in mouse embryonic neural stem cells (NSCs). This, in turn, results in altered developmental control gene expression and may form the basis for NTDs (Shyamasundar et al., 2013). Furthermore, Yang et al. have shown that high glucose increases histone H3 acetylation, which leads to NTDs. An additional study has reported that high glucose‐induced oxidative stress represses sirtuin deacetylase expression and increases histone H3 acetylation, leading to NTDs (Yu, Wu, & Yang, 2016). Additional studies have shown that acetylation of histone H3 at lysine 27 (H3K27ac) at a specific differentiation gene enhancer region is necessary for the conversion of embryonic stem cells into NSCs (Rada‐Iglesias et al., 2011). Balmer has differentiated human embryonic stem cells to neuroectodermal precursors as a model to investigate the mechanisms of action of the histone deacetylase inhibitor, Tricostation A. This study reported that Tricostation A affects the differentiation of embryonic stem cells into NSCs (Balmer et al., 2012). In addition, previous animal studies have shown that injection of the histone deacetylase inhibitor, valproic acid, or Tricostatin A induces NTDs (Copp & Greene, 2010; Murko et al., 2013). Moreover, genetic studies have found that a loss of histone acetyltransferase EP300, CREBBP, and CITED2 function may cause the occurrence of NTDs (Copp & Greene, 2010). A case–control study, including 297 spina bifida cases and 300 controls, found 37 SNPs within EP300 and CREBBP were associated with the occurrence of spina bifida (Lu et al., 2010). Taken together, these reports suggest that histone acetylation plays an important role in neural tube development. Therefore, we sought to assess histone acetylation sites and their differential expression using mass spectrometry (MS) in human NTDs.

In this study, we used the Nano‐HPLC‐MS/MS (Q Exactive HF MS) to examine histone H4 acetylation in the brains of four healthy fetuses and four fetuses with NTDs and compare acetylation sites. Furthermore, we developed a rapid MS method (LC‐ESI‐MS) to compare the histone H4 acetylation expression between NTDs and healthy control brains. Our results showed that the histone H4 acetylation expression increased in NTDs. This indicated that abnormal modification of histone H4 acetylation is a potential pathogenic mechanism in NTDs.

## 2 MATERIALS AND METHODS

### 2.1 Ethical compliance

The investigation was approved by the Committee of Medical Ethics of the Capital Institute of Pediatrics. Written informed consent was obtained from all mothers who participated in this study.

### 2.2 Subjects

Tissue with NTDs (spina bifida) and healthy control tissue were selected from Shanxi Province in northern China. Brain tissue from four fetuses with spina bifida and four healthy control fetuses (gestational age, ~20 weeks) was analyzed (Table 1). All cases were from medical abortions diagnosed with NTDs using B‐mode ultrasound. Pathological diagnosis of NTDs was performed by experienced pathologists according to the International Classification of Disease. Age‐ and sex‐matched healthy control fetuses that were aborted for nonmedical reasons were also enrolled from this region. Any fetuses displaying pathological malformations or intrauterine growth retardation were excluded from the control group.

**Table 1:** Clinical information of the eight individual fetuses

No.	Source of brain tissue	Hcy level (pmol/mg)	folate level（ng/mg）	Gender	Age
1	Normal control	3.361722	0.1109	Male	19 weeks
2	Normal control	2.166592	0.0462	Male	20 weeks
3	Normal control	4.092798	0.1093	Female	21 weeks
4	Normal control	2.060746	0.0459	Female	20 weeks
5	Open lumbosacral spina bifida	34.246276	0.1647	Male	24 weeks
6	Open thoracolumbar spine	30.88228	0.1792	Male	20 weeks
7	Closed lumbosacral spina bifida	57.575095	0.1436	Female	20 weeks
8	Open lumbar spina bifida	42.48655	0.0698	Female	26 weeks

### 2.3 Histone extraction

Histones were extracted from brain samples using acid extraction (Hake, Shechter, Dormann, & Allis, 2007). Briefly, tissue was homogenized in 10 ml lysis buffer containing 10 mM Tris‐Cl (pH 8.0), 1 mM KCl, 1.5 mM MgCl2, and 1 mM dithiothreitol (DTT). Protease and phosphatase inhibitors were added immediately before histone extraction. Nuclei were isolated by centrifugation (1,500 g for 10 min). Next, 0.2 N sulfuric acid (H_2_SO_4_) was added overnight at 4°C to isolate the histones. Following this, the supernatant was precipitated with trichloroacetic acid. Next, the histone‐containing pellets were washed with ice acetone and dissolved in distilled water. The extracted histones were stored at −80°C before analysis, as reported previously (Zhang et al., 2013).

### 2.4 In‐solution protein digestion

As described previously (Zhang et al., 2013), 40μg of extracted histones were digested. Briefly, disulfide bonds were reduced with 10mM (final concentration) DTT for 1hr at 37°C, followed by alkylation *via* 40mM (final concentration) iodoacetamide for 1hr at room temperature in the dark. The alkylation reaction was quenched with 40mM DTT for 30 min. After urea dilution to <1 M with 25mM NH_4_HCO_3_, sequence‐grade trypsin (Pierce, 90,057) was added at a ratio of 1:40 (enzyme: total protein), and proteins were digested at 37°C for 4 hr. Tryptic digestion was quenched by adding 1.0% trifluoroacetic acid. Finally, the solution was centrifuged at 13,000 g for 10 min to remove any insoluble material. The supernatant was collected and stored in 80°C for subsequent analysis.

### 2.5 Nano‐HPLC‐MS/MS

The digested samples were loaded onto 15‐cm C18 columns (particle size, 2 µm; diameter, 75 µm) using the autosampler connected to an UltiMate 3000RSLCnano system (Dionex). The peptides were eluted with a linear gradient of buffer B (0.1% formic acid in 95% ACN, v/v) from 4% to 45% in 120 min, followed by a steep increase to 90% in 5 min at a flow rate of 300 nl/min. The eluted peptides were ionized and sprayed into a Q Exactive HF mass spectrometer (Thermo Fisher Scientific) using a Nanospray Flex source in positive mode. A full mass scan (m/z 350–2000) was used at a resolution of 120,000. Twenty of the most intense ions were isolated for MS/MS analysis. The automatic gain control (AGC) target was set at 1 × 106 ions, and the maximum ion injection time (IT) was 50 ms. Source ionization parameters were optimized with the spray voltage at 2.5 kV. Other parameters were as follows: capillary temperature, 320°C; S‐Lens RF level, 50.

The MS raw data were searched using Proteome Discoverer (version 2.1.0.81) against the database of human histones, downloaded from Uniprot (http://www.uniprot.org, October 2015). The precursor mass tolerance and fragment mass tolerance are 20 ppm and 0.05 Da, respectively. Peptides were generated from the trypsin (semi) digest with up to four missed cleavage sites. Fixed modification was carbamidomethyl (C) and variable modifications were acetyl (K) (42.011 Da) and oxidation (M). Peptide spectral matches (PSMs) were validated using a percolator based on q‐values at a 1% false discovery rate (FDR). Only proteins with a minimum of two quantifiable peptides were included in our final dataset.

### 2.6 LC‐ESI‐MS analysis of histone H4

LC‐ESI‐MS analysis of the extracted histone proteins was performed using an Agilent series 1,200 pump connected to the Agilent 6,530 Q‐TOF MS with an Agilent Jet stream electrospray ionization in positive ion mode. The LC condition was as follows: mobile phase, 3% ACN/0.1% aqueous formic acid; isobaric elution was conducted by hold at 3% ACN/0.1% aqueous formic acid for 1 hr. The flow rate was 0.2 ml/min. The column was a TSK‐GEL G2000SWXL, 7.8 × 300 mm, 5‐m column with a guard column of the same packing. The injection volume was 10 μl. The ESI system employed a 3.5‐kV spray voltage (positive polarity). The drying gas (nitrogen) temperature was set at 325°C, drying gas flow at 12 L/min, nebulizer pressure at 50 psi, and Fragmentor voltage at 175 V. The mass chromatograms were recorded in total ion current (TIC) within 500 and 2,000 m/z. The deconvoluted ESI mass spectra of histones were obtained using Mass Hunter 1.0. The peak averaged mass spectra were reconstructed and the mass of the histones and their isoforms were calculated. The relative abundance of each histone isoform was derived and transformed into relative percentage amount. t test was used to compare frequencies between categorical variables and groups. Differences were considered statistically significant if *p* < .05.

### 2.7 Western blotting

Extracted histone samples (5 µg) was separated on a 12% SDS‐PAGE (Invitrogen, NW04120BOX) and transferred to nitrocellulose membranes *via* electrophoretic migration. The membrane was blocked in 5% milk/TBST (0.9%NaCl, 10mM Tris‐HCl, 0.05% Tween 20 pH 7.5) and incubated with primary antibodies: Kac (1:1,000; CST, 9,441); H3 (1:100,000; Abcam, ab213257); H4K5ac (1:2000; Abcam, ab124636); H4 (1:2000; Abcam, ab222763) overnight at 4ºC. After washing three times with TBST, the membrane was immersed in 5% milk/TBST containing horseradish peroxidase (HRP)‐conjugated secondary antibody (1:5,000; Zhongshan Jinqiao, ZB5301) for 1 h at room temperature. The immune complexes were detected using ECL (Pierce, 32,209). Results were presented as mean ± standard deviation (*SD*) from at least three independent experiments. Data analysis was performed using t test. Differences were considered significant if *p* < .05.

## 3 RESULTS

### 3.1 Sample preparation and histone extraction

**Figure 1:**
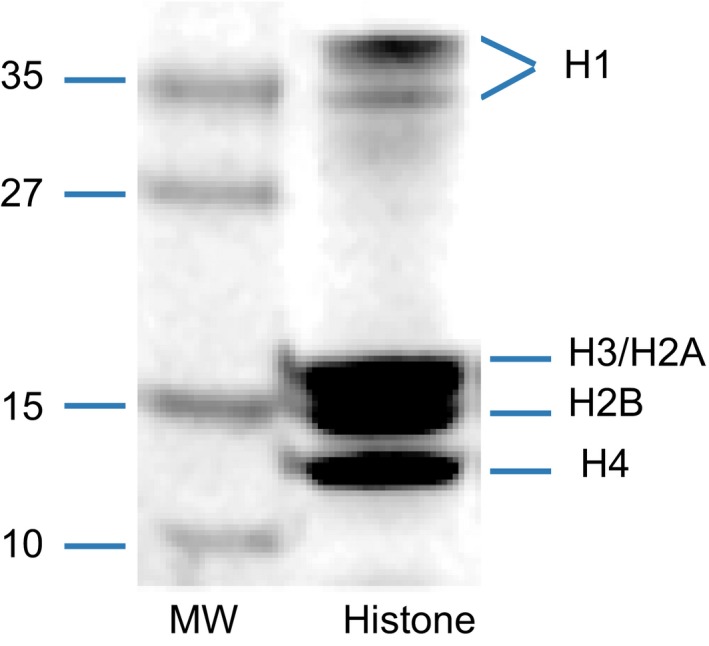
Coomassie Blue‐stained SDS gel with the extracted histone mixture for MS. The locations of the histone five major components (H1, H2a, H2b, H3, and H4) were noted. MW means molecular weight

To explore the distribution of histone acetylation in the human fetal brain, samples were analyzed using Q Exactive HF. We matched all case and control samples for age and gender. Detailed clinical information is shown in Table 1. Coomassie blue‐stained SDS gel results (Figure 1) indicated that the five major components (H1, H2a, H2b, H3, and H4) of core histone could be separated clearly from the histone extract.

### 3.2 Mass spectrometry mapping of histone acetylation in the human fetal brain

**Figure 2:**
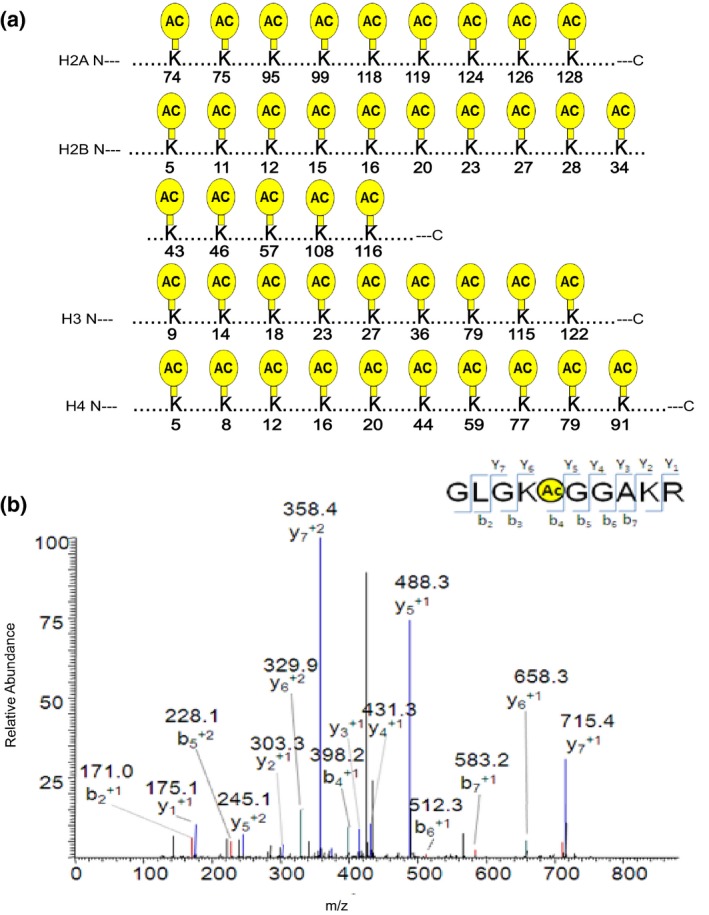
Schematic illustration of acetylation sites of histone lysine residues in human brain samples identified using HPLC‐MS/MS. (a) sites of histone acetylation detected in normal fetal brains. The yellow circle shape depicts acetylation sites in core histones (H3, H4, H2a, and H2b). (b) MS/MS spectra of a tryptic peptide ion histone H4K12 acetylated peptide (GLGKacGGAKR) in normal fetal brains. The *x* and *y* axes represent m/z and relative ion intensity, respectively. A series of b‐ and y‐type fragment ions are evident which not only provide reliable sequence information, but also indicate an unambiguous acetylation modification

In this study, a total of 43 acetylation sites were identified in healthy control human fetal brains, including K74,K75,K95,K99,K118,K119,K124,K126,K128 in H2a; K5,K11,K12,K15,K16,K20,K23,K27,K28,K34,K43,K46,K57,K108,K116 in H2b; K9,K14,K18,K23,K27,K36,K79,K115,K122 in H3; and K5,K8,K12,K16,K20,K44,K59,K77,K79,K91 in histone H4 (Figure 2a). The spectra (Figure 2b) show the representative lysine acetylation human brain H4K12 peptide GLGKacGGAKR identified by MS/MS. The series of b‐ and y‐type acetylation fragment ions provided confident sequence information and indicated an unambiguous acetylation site. Among these sites, 27 acetylation sites had been detected in humans in previous reports (Horikoshi, 2013; Kimura, 2013). However, 16 acetylation sites, including H2aK74, H2aK75, H2aK99, H2aK118, H2aK124, H2aK126, H2aK128, H2bK16, H2bK23, H2bK27, H2bK28, H2bK34, H2bK43, H2bK57, H4K44, and H4K59 were reported for the first time. All identified histone peptides of two groups were listed in Table S1 and S2. All peptides contained histone modifications that were detected on histone H4 in the four healthy fetal brains was listed in Table 2. Taken together, these data identified a novel specific histone acetylation map in the human fetal brain.

**Table 2:** Summary of acetylation peptides of histone H4 identified by Nano‐HPLC‐MS/MS in normal fetal brains

Modification Site	Peptide sequence and modification	MH + a [Da]	charge^b^	DeltaCn^c^	XCorr^d^	Confidence^e^
K5	MSGRGK_Acetyl_GGKGLGKGGAK	1,787.913622	2	0	1.927143455	High
	GK_Acetyl_GGKGLGKGGAK	1,240.702806	2	0	3.515420198	High
	K_Acetyl_GGKGLGKGGAKR	1,381.795702	2	0	2.113160849	High
	GK_Acetyl_GGKGLGKGGAKR	1,438.815599	2	0	2.742053986	High
	MSGRGK_Acetyl_GGKGLGK	1,490.771044	2	0	2.032351017	High
K8	GKGGK_Acetyl_GLGKGGAKR	1,438.814745	2	0	4.259084702	High
	GKGGK_Acetyl_GLGKGGAK	1,240.702806	2	0	3.515420198	High
	KGGK_Acetyl_GLGKGGAKR	1,381.795702	2	0	2.113160849	High
	GGK_Acetyl_GLGKGGAKR	1,211.686449	2	0	2.340593338	High
	RGKGGK_Acetyl_GLGKGGAK	1,600.866625	2	0	1.983496785	High
K12	GKGGKGLGK_Acetyl_GGAK	1,240.702806	2	0	3.515420198	High
	KGGKGLGK_Acetyl_GGAKR	1,381.795702	2	0	2.113160849	High
	GKGGKGLGK_Acetyl_GGAKR	1,438.815372	3	0	4.971486568	High
	GGKGLGK_Acetyl_GGAKR	1,211.679857	2	0	3.015662432	High
	GRGKGGKGLGK_Acetyl_GGAK	1,585.838169	3	0.0272	2.856269836	High
	GKGLGK_Acetyl_GGAKRHR	1,391.808519	2	0	2.102574348	High
	MSGRGKGGKGLGK_Acetyl_GGA	1,647.836595	2	0	2.614582539	High
K16	MSGRGKGGKGLGKGGAK_Acetyl_	1,789.964403	2	0	1.995563865	High
	GRGKGGKGLGKGGAK_Acetyl_	1,483.818394	3	0.0496	2.297608614	High
	GKGLGKGGAK_Acetyl_RHR	1,463.801561	2	0	2.216907024	High
	KGGKGLGKGGAK_Acetyl_R	1,381.795702	2	0	2.113160849	High
	GKGGKGLGKGGAK_Acetyl_R	1,438.815372	3	0	4.971486568	High
	GGKGLGKGGAK_Acetyl_R	1,211.684374	2	0	2.888950586	High
K20	K_Acety_VLRDNIQGITKPAIR	1,878.137106	4	0	5.521019936	High
K44	VK_Acetyl_RISGLIYEETRGVLKVFLENVIR	3,203.867927	5	0	2.860794067	High
K59	GVLK_Acetyl_VFLENVIR	1,428.857714	2	0	2.620787621	High
	VKRISGLIYEETRGVLK_Acetyl_VFLENVIR	3,203.867317	5	0	3.749208689	High
K77	AVTYTEHAK_Acetyl_RKTVTAMDVVYALK	2,665.357151	3	0	2.517678738	High
	AVTYTEHAK_Acetyl_RKTVTAMDVVYALKR	2,995.549595	3	0	2.991867781	High
	TYTEHAK_Acetyl_RK_Acetyl_TVTAMDVVYALKR	2,623.424717	3	0	2.651541948	High
	YTEHAK_Acetyl_RKTVTAMDVVYALKR	2,622.4202	3	0.0169	2.322541475	High
	HAK_Acetyl_RKTVTAMDVVYALKR	2,199.261814	3	0	2.458812237	High
	LENVIRDAVTYTEHAK_Acetyl_R	2,071.078647	3	0.0211	2.78817749	High
	VTYTEHAK_Acetyl_RKTVTAMDVVYALK	2,622.408481	3	0	3.066841602	High
	EHAK_Acetyl_RKTVTAMDVVYALKR	2,300.218845	3	0	2.742209196	High
	AK_Acetyl_RKTVTAMDVVYALKR	2,048.173632	2	0	1.932291985	High
	DAVTYTEHAK_Acetyl_RKTVTAMDVVYALKR	2,950.511143	3	0	3.67817688	High
K79	TYTEHAKRK_Acetyl_TVTAMDVVYALKR	2,623.424717	3	0	2.651541948	High
	AKRK_Acetyl_TVTAMDVVYALKR	2,048.173632	2	0	1.932291985	High
	KRK_Acetyl_TVTAMDVVYALK	2,009.098911	3	0	2.698174	High
	K_Acetyl_TVTAMDVVYALKR	1,636.905369	3	0	2.493818045	High
	VTYTEHAKRK_Acetyl_TVTAMDVVYALK	2,622.408481	3	0	3.066841602	High
	DAVTYTEHAKRK_Acetyl_TVTAMDVVYALKR	2,950.605442	3	0	3.611026525	High
	AVTYTEHAKRK_Acetyl_TVTAMDVVYALKR	2,995.549595	3	0	3.535665035	High
	HAKRK_Acetyl_TVTAMDVVYALKR	2,199.292393	3	0	3.398030996	High
	EHAKRK_Acetyl_TVTAMDVVYALKR	2,300.218845	3	0.0435	2.856972218	High
	AVTYTEHAKRK_Acetyl_TVTAMDVVYALK	2,665.357151	3	0	2.517678738	High
K91	TVTAMDVVYALK_Acetyl_R	1,536.852953	2	0	3.142895699	High
	KTVTAMDVVYALK_Acetyl_R	1,636.912327	3	0	4.236666203	High

a Displays the protonated monoisotopic mass of the peptides. It is the measured mass.

b ion charge; Displays the charge state of the peptide.

c DeltaCn value displays the normalized score difference between the currently selected PSM and the highest‐scoring PSM for that spectrum. Credible criteria < 0.05.

d Scores the number of fragment ions that are common to two different peptides with the same precursor mass and calculates the cross‐correlation score for all candidate peptides queried from the database.

e Confidence: Indicates a confidence level associated with the peptide sequence at the top level. The false discovery rate (FDR) is a statistical value that estimates the number of false positive identifications among all identifications found by a peptide identification search. Specifies the target false discovery rate for peptide matches of high confidence. Peptide matches that pass the filter associated with the strict FDR (0.01) indicates a high confidence.

To further assess the histone acetylation alterations associated with NTDs, we compared histone acetylation sites between the NTD and healthy control samples. These results revealed no emerging or missing acetylation sites in NTD samples compared with healthy controls. The acetylation distribution of all fetal brain tissues is shown in Table 3. This suggests there is no difference in detectable acetylated histone sites between the NTD and control samples.

**Table 3:** Acetylation distribution of eight fetal brain tissues

Protein name	Modification site	Normal control				NTDs			
		1	2	3	4	5	6	7	8
H2a	K74	●	●	●	○	●	○	●	●
	K75	○	●	○	○	●	○	●	●
	K95	●	●	●	●	●	●	●	●
	K99	●	●	●	●	●	●	●	●
	K118	○	○	●	●	○	○	○	●
	K119	●	○	●	○	●	○	●	●
	K124	●	○	●	○	●	●	●	●
	K126	●	○	●	○	●	○	○	●
	K128	●	●	○	○	○	●	○	○
H2b	K5	●	●	●	●	●	●	●	●
	K11	●	●	●	●	○	●	●	●
	K12	●	●	●	●	●	●	●	●
	K15	●	●	●	●	●	●	○	●
	K16	●	●	●	●	●	●	○	●
	K20	●	●	●	●	●	●	●	●
	K23	○	●	○	●	●	○	○	○
	K27	○	●	○	○	●	○	○	○
	K28	●	●	○	●	●	●	○	○
	K34	●	●	○	●	●	●	●	●
	K43	●	●	●	●	○	●	●	●
	K46	●	●	●	●	●	●	●	●
	K57	●	●	●	●	●	●	●	●
	K108	●	●	●	●	●	●	●	●
	K116	●	●	●	●	○	●	○	●
H3	K9	○	●	○	●	○	○	○	●
	K14	●	○	○	●	○	○	●	●
	K18	●	●	●	●	●	●	●	●
	K23	●	●	●	●	●	●	●	●
	K27	●	●	●	●	●	○	●	●
	K36	●	●	●	●	●	○	●	●
	K79	○	●	●	○	○	○	●	●
	K115	●	○	●	○	○	●	○	●
	K122	●	●	●	●	○	○	○	●
H4	K5	●	●	●	●	○	○	●	●
	K8	●	●	●	●	●	●	●	●
	K12	●	●	●	●	●	●	●	●
	K16	●	●	●	●	●	●	●	●
	K20	○	○	●	○	○	○	○	●
	K44	○	○	●	●	○	○	○	●
	K59	●	●	●	●	●	●	○	●
	K77	●	●	●	●	●	●	●	●
	K79	●	●	●	●	●	●	●	●
	K91	●	●	●	●	●	●	○	●

Note

●:detected in samples.

○: not detected in samples.

### 3.3 The histone acetylation in human fetal brain

**Figure 3:**
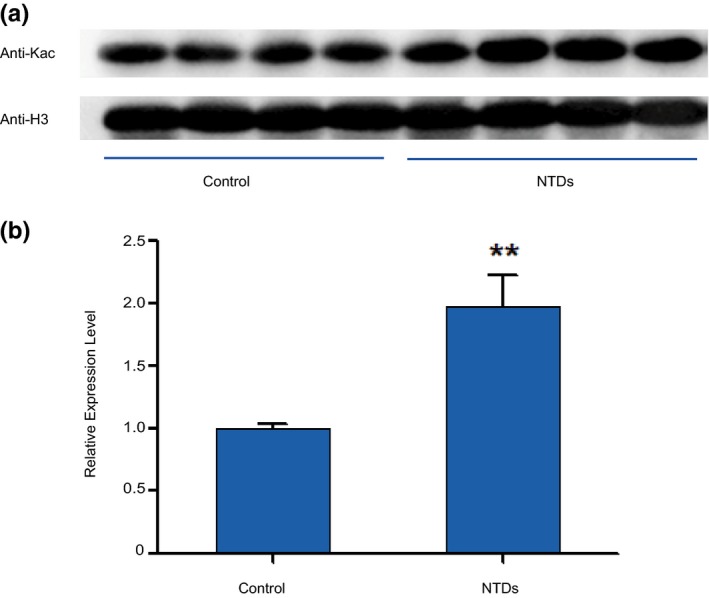
Differential levels of histone acetylation in control and NTD samples. (a) Western blot of Kac in the normal and NTD (spina bifida) samples. (b) The quantitative analysis of western blot showed that the levels of acetylation expression in NTDs are higher than in controls. The differentiation have a statistically significant (*p* < .01 compared with the control group, t test)

Although the histone acetylation sites were same in NTDs and control samples, we could not exclude any quantitative change between groups. Therefore, we performed western blotting using a specific Kac antibody and found a significantly increased expression in the NTD samples when compared with normal samples (Figure 3).

### 3.4 LC‐ESI‐MS analysis of posttranslational modifications of human brain histones

**Figure 4:**
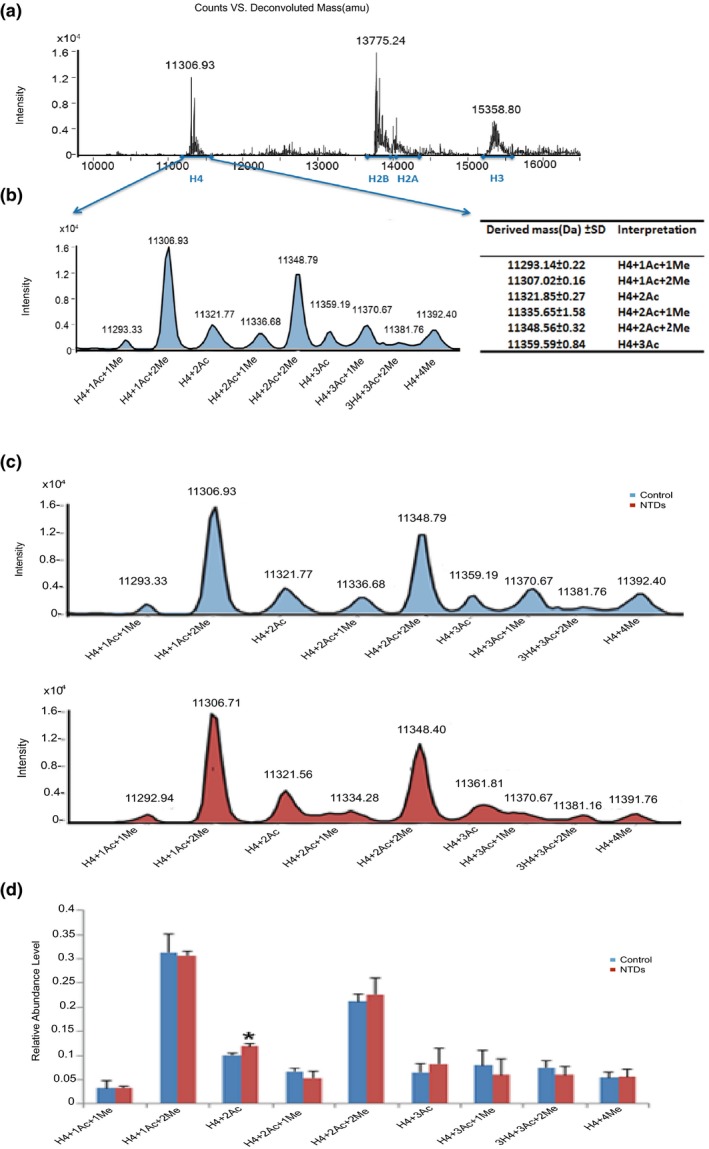
Histone modification profiles in control and NTD samples. (a) Deconvoluted ESI mass spectra of histones in Normal control fetal brains. The four peaks correspond to H4, H2b, H2a, and H3, respectively. They are marked with arrows in the spectrum. The peak of 11,306.93 Da corresponds to histone H4. (b) Reconstructed peak averaged mass spectra and the histone H4 isoforms with different acetylation was interpreted by the derived mass. H4+1AC+1Me on behalf of the H4 which have a acetylation site and a methylation site; H4+1AC+2Me on behalf the H4 which have a acetylation site and two methylation sites; H4+2AC on behalf the H4 which have two acetylation sites; H4+2AC+1Me on behalf the H4 which have two acetylation sites and a methylation site; H4+2AC+2Me on behalf the H4 which have two acetylation sites and two methylation sites; H4+3AC on behalf the H4 which have three acetylation sites. (c) Reconstructed peak averaged mass spectra of histone H4 isoforms in control and NTD samples. (d) Histograms of the quantitative analysis of histone acetylation modification along with their percentage relative abundance. (*p* < .05 compared with the control group, t test)

To evaluate the levels of acetylation in the four histone components between NTD and control samples, we next developed a rapid MS method (LC‐ESI‐MS). The extracted histones were separated using a molecular sieve chromatography column, which showed four major peaks, including H3, H4, H2a, and H2b (Figure 4a). The assignments were based on the masses predicted from known amino acid sequences and allowing for posttranslational modifications (Bonenfant, Coulot, Towbin, Schindler, & Oostrum, 2006; Zhang et al., 2004). Compared with the traditional method using a C4 or C18 column, the elution pattern was consistent and reproducible with similar results obtained for other human cell lines, such as Jurkat cells or bovine thymus. Extracted histone mixtures containing H2a, H2b, H3, and H4 were clearly separated (Figure 4a). The peak marked with 11,306.93 was identified as histone H4 (Figure 4a) by the deconvoluted ESI mass spectra analyzed by an on‐line ion trap with Mag Tran 1.0 software. Furthermore, Histone H4 with a series of acetylation sites (Figure 4b) was identified by mass analysis. Each histone protein modification was indicated by derived molecular mass calculated by software, as reported in Figure 4b, together with their percentage relative abundance. We detected the percentage relative abundance of histone H4 acetylation in NTDs and healthy controls samples (Figure 4c). The relative abundance of all samples was shown in Table 4. Among the nine types that were identified (H4+1AC+1Me, H4+1AC+2Me, H4+2AC, H4+2AC+1Me, H4+2AC+2Me, H4+3AC, H4+3AC+1Me, H4+3AC+2Me, H4+4AC), there was a significant difference in H4+2AC relative abundance between healthy controls and NTD samples. These results showed that the level of H4 with two acetylation was significantly increased in NTD samples compared with healthy controls (Figure 4d).

**Table 4:** The relative abundance of peptides detected from the four normal fetal brains and four NTDs

No^a^	H4 + 1AC + 1Me	H4 + 1AC + 2Me	H4 + 2AC	H4 + 2AC + 1Me	H4 + 2AC + 2Me	H4 + 3AC	H4 + 3AC + 1Me	H4 + 3AC + 2Me	H4 + 4AC
1	902	3,031	1,286	1,096	2,424	1,427	571	888	1,028
2	315	2,986	768	529	1813	636	307	412	329
3	288	3,348	1,193	818	2,625	675	1,159	1,043	769
4	403	3,437	1,263	779	2,256	702	1,247	1,029	678
5	366	3,100	1,230	290	2,679	1,184	244	483	483
6	362	3,015	1,248	723	1875	532	932	910	757
7	249	2,664	906	403	1702	424	785	570	579
8	301	2,907	1,179	614	2,419	1,044	314	348	334

a The number is the same as the clinical information number of the eight individual fetus.

### 3.5 H4K5ac expression validation

**Figure 5:**
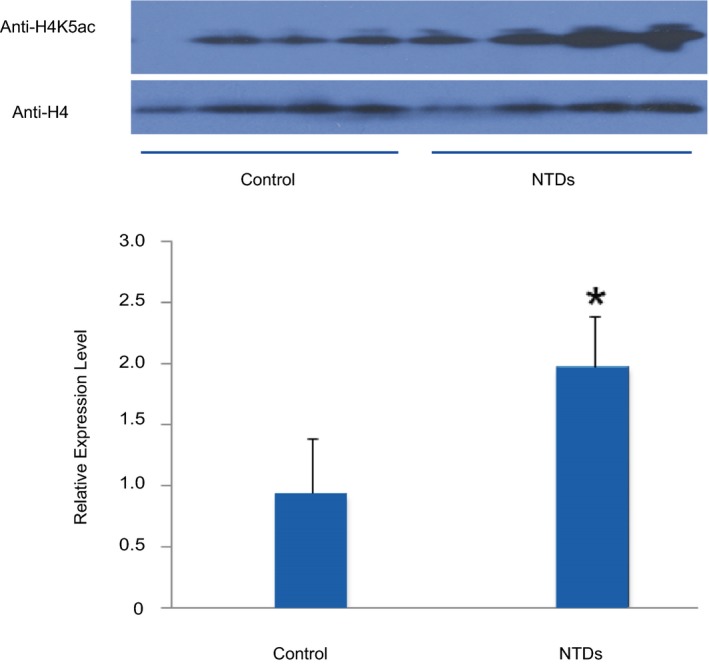
Histone acetylation comparation of H4K5ac in control and NTD samples**.** (a) Western blot of H4K5ac in the normal and NTD (spina bifida) samples. (b) Histograms of the quantitative analysis of H4K5ac expression using western blot (*p* < .05 compared with the control group, t test)

Based on previously reported alterations in H4K5ac in a mouse embryo model of maternal diabetes with NTDs (Salbaum & Kappen, 2012), we sought to validate the alterations in H4K5ac in NTDs and healthy controls in this study. Therefore, we performed western blotting using a specific anti‐H4K5ac antibody. These results indicated that H4K5ac expression was detected in all healthy control samples, but were significantly higher in the NTD brain samples (Figure 5).

In summary, histone H4K5ac levels in brain samples with NTDs were significantly when compared with healthy controls**.** These results indicated quantitative difference in histone H4 acetylation in human NTDs.

## 4 DISCUSSION

Histone posttranslational modifications play an important role in the dynamics of chromatin structure and function. However, no comprehensive analysis of histone acetylation has been reported at the protein level in the developing human brain. In this study, we used a strategy combining mass spectrometric techniques (nano‐HPLC‐MS/MS and LC‐ESI‐MS), molecular sieve separation, and enzymatic digestions to characterize histone acetylation in human fetal samples. Taken together, we obtained a comprehensive overview of the human brain histone acetylation, in particular associated with histone H4 acetylation. Using two MS methods, we concluded that there is no difference in detectable of histone acetylation sites between the NTDs and control samples. But the acetylation level of histone H4 in NTDs increased. Here, we showed that the quantity of histone H4 acetylation was important for normal brain development; an increase in histone H4 acetylation was linked to NTDs.

Histone acetylation regulates many cellular processes, such as gene expression. Acetylation marks within the histone N terminal tails and globular domains can selectively make the structure of certain chromatin regions from tightly dispersed, facilitating the binding of transcription factors to DNA, thereby promoting the transcription of certain genes and enhancing the expression level of genes. Acetylated lysine within the H4 tails (K5, K8, and K12 of H4) and globular domains (K56 of H3 and K91 of H4) appear to serve important, albeit redundant, roles in chromatin assembly (Ma, Wu, Altheim, Schultz, & Grunstein, 1998; Ye et al., 2005). Another role for histone acetylation is the regulation of chromatin folding. Acetylation of the histone H4 tail plays a prominent role in determining the ability of chromatin to fold into higher‐order structures (Chang & Takada, 2016; Luger, Mader, Richmond, Sargent, & Richmond, 1997). Acetylation promotes a more open chromatin configuration by decreasing the interactions between nucleosomes. This releases the histone tails from linker DNA and reduces fiber–fiber interactions. In addition, histone acetylation regulates the formation of heterochromatin; acetylation of H4 lysine 16 is important to regulate chromatin accessibility(Zhang, Erler, & Langowski, 2017) and spread heterochromatin components, whereas acetylation of this site serves as a barrier to this spreading (Liou, Tanny, Kruger, Walz, & Moazed, 2005). Finally, histone acetylation is critical for gene transcription (Chen, Zhao, & Zhao, 2015; Chen et al., 2019). Acetylation promotes transcription by favoring a more open chromatin conformation that permits binding of the transcriptional machinery and by directly serving as recognition sites for factors that promote transcription (Shahbazian & Grunstein, 2007; Wilson & Merkenschlager, 2006).

Histone acetylation has a close association with the occurrence of NTDs. It is crucial in gene transcription and plays a key role in late brain development (Antonio & Silvia, 2013). Furthermore, it is dynamically regulated by several classes of histone deacetylases (HDACs) and families of histone acetyltransferases (HATs), such as P300, CBP, and GCN5 (Oc, 2012). These act on targeted regions of chromatin to regulate specific gene transcription. One study reported that Mark2 depletion reduces *Dvl* gene expression and interrupts neural stem cell (NSCs) growth and differentiation in mouse cell line, which are likely to be mediated through a decrease in class IIa HDAC phosphorylation and reduced H3K4ac and H3K27ac occupancies at the *Dvl1/2* promoters(Chen et al., 2017). High glucose is a significant risk factor for NTDs. Bai et al. have reported that maternal diabetes leads to an increase in H4K5/K8/K12/K16 acetylation levels, and that the CBP selective inhibitor, C646, could efficiently prevent an increase or emergence of histone H4 acetylation and neuroepithelial cell proliferation (Bai et al., 2018). Furthermore, a deletion in the neuronal *Nap1l2* (nucleosome assembly protein 1‐like 2) gene in mice leads to an increase in acetylated histone H3K9/14 at the *Cdkn1c* transcription site and causes NTDs (Attia, Rachez, De Pauw, Avner, & Rogner, 2007). Taken together with our results, the alterations in histone acetylation influence the gene transcription in a genome‐wide profile, and histone acetylation is associated with the occurrence of NTDs. Moreover, gene‐specific changes in histone H4 acetylation patterns may be a key early step in the pathological processes triggered by status epileptics (Huang, Doherty, & Dingledine, 2002). Previous research (Cho, Kim, Kim, & Sun, 2011) has found that the level of histone H4 acetylation is highly dynamic and tightly linked to neuronal type and differentiation stage. Furthermore, histone H4 acetylation contributes to the action of *IGF‐I* on gene expression in the mammalian central nerve system (Sun & D’Ercole, 2006). Histone modifications have complex interactions. H4K5ac results in decreased arginine methylation by PRMT1, PRMT3, and PRMT8 (Fulton, Zhang, He, Ho, & Zheng, 2017). Taken together, this indicates that inordinate histone H4 acetylation may be involved in etiology of NTDs.

## 5 CONCLUSION

In summary, this study presents a comprehensive map of histone H4 modifications in the human fetal brain and provides experimental evidence supporting a relationship between histone H4 acetylation and NTDs. Combined with our previous work that shows that aberrant histone methylation is involved in NTDs (Zhang et al., 2013), these results confirm that aberrant histone modification during early pregnancy is associated with the occurrence of NTDs.

## CONFLICT OF INTEREST

All authors declared that no competing interests existed.
