# Supplementary material for: Identification of histone acetylation markers in human fetal brains and increased H4K5ac expression in neural tube defects
Source: Mol Genet Genomic Med. 2019 Oct 14;7(12):e1002. doi: 10.1002/mgg3.1002 (PMC6900389; doi:10.1002/mgg3.1002)
Supplement: Supplementary file 1 [file MGG3-7-e1002-s001.docx]

Supplementary table 1 Histone peptides including acetylation identified by Nano-HPLC-MS/MS in control samples

| **Protein Name** | **Modification Site** | **Peptide sequence and modification** | **MH+^a^** | **Confidence**^b^ |
| --- | --- | --- | --- | --- |
| H2a | K74 | AERVGAGAPVYLAAVLEYLTAEILELAGNAARDNK_Acetyl_K | 3841.11 | High |
|  |  | VGAGAPVYLAAVLEYLTAEILELAGNAARDNK_Acetyl_KTRII | 3996.27 | High |
|  |  | AAVLEYLTAEILELAGNAARDNK_Acetyl_K | 2615.42 | High |
|  |  | AVLEYLTAEILELAGNAARDNK_Acetyl_KTR | 2843.47 | High |
|  |  | ELAGNAARDNK_Acetyl_KTR | 1787.93 | High |
|  | K75 | VGAGAPVYLAAVLEYLTAEILELAGNAARDNKK_Acetyl_TRII | 3996.27 | High |
|  |  | AVLEYLTAEILELAGNAARDNKK_Acetyl_TR | 2843.47 | High |
|  | K95 | DEELNK_Acetyl_LLGKVTIAQGGVLPNIQAVLLPK | 3286.87 | High |
|  |  | NDEELNK_Acetyl_LLGKVTIAQGGVLPNIQAVLLPK | 3268.85 | High |
|  |  | NDEELNK_Acetyl_LLGK | 1314.69 | High |
|  |  | HLQLAIRNDEELNK_Acetyl_LLGK | 2174.16 | High |
|  |  | NDEELNK_Acetyl_LLGKVTIAQGGVLPNIQAVLLPK | 3268.85 | High |
|  |  | HLQLAIRNDEELNK_Acetyl_LLGKVTIAQGGVLPNIQAVLLP | 3972.24 | High |
|  |  | LQLAVRNDEELNK_Acetyl_LLGGVTIAQGGVLPNIQAVLLPK | 3949.29 | High |
|  |  | QLAIRNDEELNK_Acetyl_LLGKVTIAQGGVLPNIQAVLLPK | 3996.27 | High |
|  |  | ELNK_Acetyl_LLGKVTIAQGGVLPNIQAVLLPK | 2910.73 | High |
|  | K99 | ELNKLLGK_Acetyl_VTIAQGGVLPNIQAVLLPK | 2910.73 | High |
|  |  | HLQLAIRNDEELNKLLGK_Acetyl_VTIAQGGVLPNIQAVLLP | 3972.24 | High |
|  |  | LQLAIRNDEELNKLLGK_Acetyl_VTIAQGGVLPNIQAVLLPK | 3949.28 | High |
|  |  | QLAIRNDEELNKLLGK_Acetyl_VTIAQGGVLPNIQAVLLPK | 3996.27 | High |
|  |  | NDEELNKLLGK_Acetyl_VTIAQGGVLPNIQAVLLPK | 3268.85 | High |
|  |  | DEELNKLLGK_Acetyl_VTIAQGGVLPNIQAVLLPK | 3286.88 | High |
|  |  | ELNKLLGK_Acetyl_VTIAQGGVLPNIQAVLLPK | 2910.73 | High |
|  |  | LGK_Acetyl_VTIAQGGVLPNIQAVLLPK | 2271.38 | High |
|  |  | NDEELNKLLGK_Acetyl_VTIAQGGVLPNIQAVLLPK | 3268.85 | High |
|  |  | GK_Acetyl_VTIAQGGVLPNIQAVLLPK | 2158.31 | High |
|  | K118 | AQGGVLPNIQAVLLPK_Acetyl_KTESQKTK | 2806.60 | High |
|  |  | PNIQAVLLPK_Acetyl_KTESQKTKSK | 2366.29 | High |
|  |  | VTIAQGGVLPNIQAVLLPK_Acetyl_KTESQKT | 2977.65 | High |
|  | K119 | PNIQAVLLPKK_Acetyl_TESQKTKSK | 2366.29 | High |
|  |  | VTIAQGGVLPNIQAVLLPKK_Acetyl_TESQKT | 2977.65 | High |
|  |  | VTIAQGGVLPNIQAVLLPKK_Acetyl_TESQK | 2760.57 | High |
|  | K124 | KTESQK_Acetyl_TK | 991.53 | High |
|  |  | VTIAQGGVLPNIQAVLLPKKTESQK_Acetyl_ | 2760.57 | High |
|  |  | VTIAQGGVLPNIQAVLLPKKTESQK_Acetyl_T | 2977.65 | High |
|  |  | AQGGVLPNIQAVLLPKKTESQK_Acetyl_TKSK | 2875.62 | High |
|  |  | GVLPNIQAVLLPKKTESQK_Acetyl_TKSK | 2705.55 | High |
|  |  | PNIQAVLLPKKTESQK_Acetyl_TKSK | 2366.29 | High |
|  |  | PNIQAVLLPKKTESQKTK_Acetyl_SK | 2366.29 | High |
|  | K128 | PNIQAVLLPKKTESQKTKSK_Acetyl_ | 2366.29 | High |
| H2b | K5 | PEPTK_Acetyl_SAPAPKKGSK | 1634.93 | High |
|  |  | PTK_Acetyl_SAPAPK | 980.55 | High |
|  |  | PVK_Acetyl_SAPVPK | 964.58 | High |
|  | K11 | KSAPVPK_Acetyl_K | 896.56 | High |
|  |  | PTKSAPAPK_Acetyl_ | 980.55 | High |
|  |  | SAPAPK_Acetyl_KGSKKAVTK | 1665.96 | High |
|  |  | SAPAPK_Acetyl_KGSKK | 1224.70 | High |
|  | K12 | PTKSAPAPKK_Acetyl_GSK | 1494.83 | High |
|  |  | SAPAPKK_Acetyl_GSKK | 1224.70 | High |
|  |  | SAPAPKK_Acetyl_GSKKAVTK | 1665.96 | High |
|  |  | K_Acetyl_GSKKAVTKAQ | 1441.84 | High |
|  | K15 | PTKSAPAPKKGSK_Acetyl_ | 1494.83 | High |
|  |  | SAPAPKKGSK_Acetyl_K | 1224.70 | High |
|  |  | SAPAPKKGSK_Acetyl_KAVTK | 1665.96 | High |
|  |  | GSK_Acetyl_KAVTKAQKK | 1399.83 | High |
|  |  | GSK_Acetyl_KAVTKAQK | 1271.74 | High |
|  |  | KGSK_Acetyl_KAVTKAQK | 1441.84 | High |
|  | K16 | KGSKK_Acetyl_AVTKAQK | 1749.92 | High |
|  |  | SAPAPKKGSKK_Acetyl_VTK | 1665.96 | High |
|  |  | GSKK_Acetyl_AVTKAQKK | 1399.83 | High |
|  |  | K_Acetyl_AVTKAQKK | 1127.68 | High |
|  |  | GSKK_Acetyl_AVTKAQK | 1271.74 | High |
|  | K20 | AVTK_Acetyl_AQKK | 915.56 | High |
|  |  | TK_Acetyl_AQKK | 745.46 | High |
|  |  | GSKKAVTK_Acetyl_AQKK | 1399.83 | High |
|  |  | GSKKAVTK_Acetyl_AQK | 1271.74 | High |
|  |  | KAVTK_Acetyl_AQKK | 1127.68 | High |
|  |  | KGSKKAVTK_Acetyl_AQK | 1441.84 | High |
|  | K23 | KAVTKAQK_Acetyl_K | 1127.68 | High |
|  | K27 | KDGK_Acetyl_KRK | 915.57 | High |
|  | K28 | KDGKK_Acetyl_R | 773.46 | High |
|  |  | KDGKK_Acetyl_RK | 915.57 | High |
|  | K34 | K_Acetyl_ESYSVYVYKVLKQVHPDTGISSK | 3013.61 | High |
|  |  | RK_Acetyl_EYSVYVYKVLKQVHPDTGISSK | 2995.55 | High |
|  |  | SRK_Acetyl_ESYSVYVYK | 1578.81 | High |
|  |  | K_Acetyl_ESYSVYVYKVLKQVHPDTGISSKAMGIMNSFVNDIFER | 4740.40 | High |
|  |  | K_Acetyl_ESYSVYVYK | 1307.67 | High |
|  | K43 | KESYSIYVYK_Acetyl_VLKQVHPDTGISSKAMGIMNSFVNDIFER | 4726.41 | High |
|  |  | RKESYSVYVYK_Acetyl_VLKQVHPDTGISSK | 2995.55 | High |
|  |  | SYSVYVYK_Acetyl_VLKQVHPDTGISSK | 2626.32 | High |
|  |  | SYSVYVYK_Acetyl_VLKQVHPDTGISSKAMGIMNSFVNDIFER | 4481.27 | High |
|  |  | YVYK_Acetyl_VLKQVHPDTGISSK | 2118.18 | High |
|  |  | VYK_Acetyl_VLKQVHPDTGISSKAMGIMNSFVNDIFER | 3707.97 | High |
|  |  | YK_Acetyl_VLKQVHPDTGISSKAMGIMNSFVNDIFER | 3666.82 | High |
|  |  | VYK_Acetyl_VLKQVHPDTGISSKAMGIMNSFVNDIFER | 3693.97 | High |
|  |  | K_Acetyl_VLKQVHPDTGISSKAMGIMNSFVNDIFER | 3577.75 | High |
|  |  | KESYSVYVYK_Acetyl_VLK | 1647.90 | High |
|  |  | KESYSIYVYK_Acetyl_VLKQVHPDTGISSK | 3013.62 | High |
|  | K46 | SYSVYVYKVLK_Acetyl_QVHPDTGISSK | 2626.32 | High |
|  |  | VYKVLK_Acetyl_QVHPDTGISSKAMGIMNSFVNDIFER | 3708.00 | High |
|  |  | KESYSVYVYKVLK_Acetyl_QVHPDTGISSKAMGIMNSFVNDIFER | 4740.41 | High |
|  |  | KVLK_Acetyl_QVHPDTGISSKAMGIMNSFVNDIFER | 3577.75 | High |
|  |  | VLK_Acetyl_QVHPDTGISSKAMGIMNSFVNDIFER | 3291.69 | High |
|  |  | K_Acetyl_QVHPDTGISSKAMGIMNSFVNDIFER | 3095.44 | High |
|  |  | VLK_Acetyl_QVHPDTGISSK | 1550.86 | High |
|  |  | SYSVYVYKVLK_Acetyl_QVHPDTGISSKAMGIMNSFVNDIFER | 4481.27 | High |
|  |  | YVYKVLK_Acetyl_QVHPDTGISSK | 2118.19 | High |
|  |  | VLK_Acetyl_QVHPDTGISSKAMGIMNSFVNDIFER | 3291.67 | High |
|  |  | YKVLK_Acetyl_QVHPDTGISSKAMGIMNSFVNDIFER | 3666.82 | High |
|  | K57 | VYKVLKQVHPDTGISSK_Acetyl_AMGIMNSFVNDIFER | 3708.00 | High |
|  |  | GISSK_Acetyl_AMGIMNSFVNDIFER | 2258.10 | High |
|  |  | SSK_Acetyl_AMGIMNSFVNDIFER | 2104.01 | High |
|  |  | SK_Acetyl_AMGIMNSFVNDIFER | 2032.93 | High |
|  |  | YKVLKQVHPDTGISSK_Acetyl_AMGIMNSFVNDIFER | 3666.82 | High |
|  |  | VLKQVHPDTGISSK_Acetyl_AMGIMNSFVNDIFER | 3291.67 | High |
|  |  | KESYSVYVYKVLKQVHPDTGISSK_Acetyl_AMGIMNSFVNDIFER | 4740.41 | High |
|  |  | KVLKQVHPDTGISSK_Acetyl_AMGIMNSFVNDIFER | 3577.75 | High |
|  |  | SYSVYVYKVLKQVHPDTGISSK_Acetyl_AMGIMNSFVNDIFER | 4483.28 | High |
|  |  | QVHPDTGISSK_Acetyl_AMGIMNSFVNDIFER | 2935.41 | High |
|  |  | VHPDTGISSK_Acetyl_AMGIMNSFVNDIFER | 2807.37 | High |
|  |  | KQVHPDTGISSK_Acetyl_AMGIMNSFVNDIFER | 3095.44 | High |
|  |  | TGISSK_Acetyl_AMGIMNSFVNDIFER | 2375.16 | High |
|  | K108 | LPGELAK_Acetyl_HAVSEGTK | 1578.85 | High |
|  |  | LLLPGELAK_Acetyl_HAVSEGTK | 1805.02 | High |
|  |  | PGELAK_Acetyl_HAVSEGTK | 1465.76 | High |
|  | K116 | LLLPGELAKHAVSEGTK_Acetyl_A | 1962.05 | High |
|  |  | LLLPGELAKHAVSEGTK_Acetyl_ | 1819.04 | High |
|  |  | LAKHAVSEGTK_Acetyl_AVTKYTSSK | 2162.19 | High |
|  |  | LPGELAKHAVSEGTK_Acetyl_ | 1620.86 | High |
|  |  | PGELAKHAVSEGTK_Acetyl_ | 1507.78 | High |
| H3 | K9 | RK_Acetyl_STGGK | 789.45 | High |
|  |  | KQTARK_Acetyl_STGGK | 1203.68 | High |
|  |  | K_Acetyl_STGGKAPR | 985.54 | High |
|  |  | QTARK_Acetyl_STGGKAPR | 1499.77 | High |
|  |  | KQTARK_Acetyl_STGGKAPR | 1569.88 | High |
|  | K14 | KQTARKSTGGK_Acetyl_APR | 1655.92 | High |
|  |  | KSTGGK_Acetyl_APR | 985.54 | High |
|  | K18 | K_Acetyl_QLATK_Acetyl_AA | 914.53 | High |
|  |  | K_Acetyl_QLATKAAR | 1070.63 | High |
|  |  | ARKSTGGKAPRK_Acetyl_ | 1514.86 | High |
|  | K23 | KQLATK_Acetyl_AAR | 1028.63 | High |
|  |  | KQLATK_Acetyl_AA | 914.53 | High |
|  |  | KQLATK_Acetyl_AARKSAPSTGGVK | 2143.14 | High |
|  |  | ATK_Acetyl_AARKSAPSTGGVKKPHR | 2132.22 | High |
|  |  | K_Acetyl_AARKSAPSTGGVKKPHR | 2132.22 | High |
|  | K27 | ATKAARK_Acetyl_SAPSTGGVKKPHR | 2304.24 | High |
|  |  | AARK_Acetyl_SAPATGGVKK | 1483.82 | High |
|  |  | AARK_Acetyl_SAPSTGGVKKPH | 1675.97 | High |
|  |  | QLATKAARK_Acetyl_SAP | 1325.76 | High |
|  |  | KAARK_Acetyl_SAPSTGGVKKPHR | 1988.14 | High |
|  |  | K_Acetyl_SAPSTGGVKKPHRYRPGTV | 2249.29 | High |
|  |  | AARK_Acetyl_SAPATGGVKKPHRYRP | 2318.25 | High |
|  |  | AARK_Acetyl_SAPSTGGVKKPHRYRPG | 2319.32 | High |
|  | K36 |  |  | High |
|  |  | KSAPSTGGVK_Acetyl_KPHR | 1577.84 | High |
|  |  | ATKAARKSAPSTGGVK_Acetyl_KPHR | 2132.25 | High |
|  |  | SAPATGGVK_Acetyl_KPHRYRPGT | 1922.05 | High |
|  |  | KAARKSAPSTGGVK_Acetyl_KPHR | 2032.14 | High |
|  |  | KSAPATGGVKK_Acetyl_PHR | 1519.82 | High |
|  | K79 | FK_Acetyl_TDLRFQSSAVMALQEACEAYLVGLFEDTNLCAIHAK | 4403.23 | High |
|  |  | EIAQDFK_Acetyl_TDLRFQSAAI | 2009.03 | High |
|  | K115 | FEDTNLCAIHAK_Acetyl_RVTIMPKDIQLAR | 2939.50 | High |
|  |  | CAIHAK_Acetyl_RVTIMPKDIQLAR | 2279.22 | High |
|  |  | AK_Acetyl_RVTIMPKDIQLARR | 1968.15 | High |
|  | K122 | TIMPK_Acetyl_DIQLARR | 1497.86 | High |
|  |  | DLRFQSAAIGALQEASEAYLVGLFEDTNLCAIHAKRVTIMPK_Acetyl_ | 4905.54 | High |
|  |  | RVTIMPK_Acetyl_DIQLAR | 1598.93 | High |
| H4 | K5 | MSGRGK_Acetyl_GGKGLGKGGAK | 1787.91 | High |
|  |  | GK_Acetyl_GGKGLGKGGAK | 1240.70 | High |
|  |  | K_Acetyl_GGKGLGKGGAKR | 1381.80 | High |
|  |  | GK_Acetyl_GGKGLGKGGAKR | 1438.81 | High |
|  |  | MSGRGK_Acetyl_GGKGLGK | 1490.77 | High |
|  | K8 | GKGGK_Acetyl_GLGKGGAKR | 1438.81 | High |
|  |  | GKGGK_Acetyl_GLGKGGAK | 1240.70 | High |
|  |  | KGGK_Acetyl_GLGKGGAKR | 1381.80 | High |
|  |  | GGK_Acetyl_GLGKGGAKR | 1211.68 | High |
|  |  | RGKGGK_Acetyl_GLGKGGAK | 1600.87 | High |
|  | K12 | GKGGKGLGK_Acetyl_GGAK | 1240.70 | High |
|  |  | KGGKGLGK_Acetyl_GGAKR | 1381.80 | High |
|  |  | GKGGKGLGK_Acetyl_GGAKR | 1438.81 | High |
|  |  | GGKGLGK_Acetyl_GGAKR | 1211.68 | High |
|  |  | GRGKGGKGLGK_Acetyl_GGAK | 1585.84 | High |
|  |  | GKGLGK_Acetyl_GGAKRHR | 1391.81 | High |
|  |  | MSGRGKGGKGLGK_Acetyl_GGA | 1647.84 | High |
|  | K16 | MSGRGKGGKGLGKGGAK_Acetyl_ | 1789.96 | High |
|  |  | GRGKGGKGLGKGGAK_Acetyl_ | 1483.82 | High |
|  |  | GKGLGKGGAK_Acetyl_RHR | 1463.80 | High |
|  |  | KGGKGLGKGGAK_Acetyl_R | 1381.80 | High |
|  |  | GKGGKGLGKGGAK_Acetyl_R | 1438.81 | High |
|  |  | GGKGLGKGGAK_Acetyl_R | 1211.68 | High |
|  | K20 | K_Acetyl_VLRDNIQGITKPAIR | 1878.14 | High |
|  | K44 | VK_Acetyl_RISGLIYEETRGVLKVFLENVIR | 3203.87 | High |
|  | K59 | GVLK_Acetyl_VFLENVIR | 1428.86 | High |
|  |  | VKRISGLIYEETRGVLK_Acetyl_VFLENVIR | 3203.88 | High |
|  | K77 | AVTYTEHAK_Acetyl_RKTVTAMDVVYALK | 2665.36 | High |
|  |  | AVTYTEHAK_Acetyl_RKTVTAMDVVYALKR | 2995.55 | High |
|  |  | TYTEHAK_Acetyl_RK_Acetyl_TVTAMDVVYALKR | 2623.42 | High |
|  |  | YTEHAK_Acetyl_RKTVTAMDVVYALKR | 2622.42 | High |
|  |  | HAK_Acetyl_RKTVTAMDVVYALKR | 2199.26 | High |
|  |  | LENVIRDAVTYTEHAK_Acetyl_R | 2071.08 | High |
|  |  | VTYTEHAK_Acetyl_RKTVTAMDVVYALK | 2622.41 | High |
|  |  | EHAK_Acetyl_RKTVTAMDVVYALKR | 2300.22 | High |
|  |  | AK_Acetyl_RKTVTAMDVVYALKR | 2048.17 | High |
|  |  | DAVTYTEHAK_Acetyl_RKTVTAMDVVYALKR | 2950.51 | High |
|  | K79 | TYTEHAKRK_Acetyl_TVTAMDVVYALKR | 2623.42 | High |
|  |  | AKRK_Acetyl_TVTAMDVVYALKR | 2048.17 | High |
|  |  | KRK_Acetyl_TVTAMDVVYALK | 2009.10 | High |
|  |  | K_Acetyl_TVTAMDVVYALKR | 1636.91 | High |
|  |  | VTYTEHAKRK_Acetyl_TVTAMDVVYALK | 2622.41 | High |
|  |  | DAVTYTEHAKRK_Acetyl_TVTAMDVVYALKR | 2950.60 | High |
|  |  | AVTYTEHAKRK_Acetyl_TVTAMDVVYALKR | 2995.55 | High |
|  |  | HAKRK_Acetyl_TVTAMDVVYALKR | 2199.26 | High |
|  |  | EHAKRK_Acetyl_TVTAMDVVYALKR | 2300.22 | High |
|  |  | AVTYTEHAKRK_Acetyl_TVTAMDVVYALK | 2665.36 | High |
|  | K91 | TVTAMDVVYALK_Acetyl_R | 1536.85 | High |
|  |  | KTVTAMDVVYALK_Acetyl_R | 1636.91 | High |

a: Displays the protonated monoisotopic mass of the peptides. It is the measured mass.

b: Confidence：Indicates a confidence level associated with the peptide sequence at the top level. The false discovery rate (FDR) is a statistical value that estimates the number of false positive identifications among all identifications found by a peptide identification search. Specifies the target false discovery rate for peptide matches of high confidence. Peptide matches that pass the filter associated with the strict FDR ( 0.01) indicates a high-confidence.

Supplementary table 2 Histone peptides including acetylation identified by Nano-HPLC-MS/MS in NTDs samples

| **Protein Name** | **Modification Site** | **Peptide sequence and modification** | **MH+^a^** | **Confidence**^b^ |
| --- | --- | --- | --- | --- |
| H2a | K13 | K_acetyl_AKSRSSR | 1061.55 | High |
|  | K74 | AERVGAGAPVYLAAVLEYLTAEILELAGNAARDNK_Acetyl_KTR | 4258.34 | High |
|  |  | PVYLAAVLEYLTAEILELAGNAARDNK_Acetyl_KTR | 3400.86 | High |
|  | K75 | PVYLAAVLEYLTAEILELAGNAARDNK_Acetyl_KTR | 3400.86 | High |
|  | K95 | DEELNK_Acetyl_LLGKVTIAQGGVLPNIQAVLLPK | 3286.87 | High |
|  |  | HLQLAIRNDEELNK_Acetyl_LLGKVTIAQGGVLPNIQAVLLPK | 4086.38 | High |
|  |  | NDEELNK_Acetyl_LLGKVTIAQGGVLPNIQAVLLPK | 3268.85 | High |
|  |  | HLQLAIRNDEELNK_Acetyl_LLGK | 2174.16 | High |
|  |  | HLQLAIRNDEELNK_Acetyl_LLGKVTIAQ | 2744.53 | High |
|  |  | NDEELNK_Acetyl_LLGKVTIAQGGVLPNIQAVLLPK | 3268.85 | High |
|  |  | LQLAVRNDEELNK_Acetyl_LLGGVTIAQGGVLPNIQAVLLPK | 3949.27 | High |
|  |  | QLAIRNDEELNK_Acetyl_LLGKVTIAQGGVLPNIQAVLLPK | 3996.27 | High |
|  | K99 | NDEELNKLLGK_Acetyl_VTIAQGGVLPNIQAVLLPK | 3268.85 | High |
|  |  | HLQLAIRNDEELNKLLGK_Acetyl_VTIAQ | 2744.53 | High |
|  |  | HLQLAIRNDEELNKLLGK_Acetyl_ | 2174.17 | High |
|  |  | LQLAIRNDEELNKLLGK_Acetyl_VTIAQGGVLPNIQAVLLPK | 3949.28 | High |
|  |  | QLAIRNDEELNKLLGK_Acetyl_VTIAQGGVLPNIQAVLLPK | 3996.27 | High |
|  |  | DEELNKLLGK_Acetyl_VTIAQGGVLPNIQAVLLPK | 3286.88 | High |
|  |  | LGK_Acetyl_VTIAQGGVLPNIQAVLLPK | 2271.38 | High |
|  | K118 | VTIAQGGVLPNIQAVLLPK_Acetyl_KTESHHK | 2848.64 | High |
|  | K119 | VTIAQGGVLPNIQAVLLPKK_Acetyl_TESHHK | 2848.64 | High |
|  |  | AQGGVLPNIQAVLLPKK_Acetyl_TESQKTK | 2806.60 | High |
|  |  | AQGGVLPNIQAVLLPKK_Acetyl_TESQKTKSK | 3065.61 | High |
|  | K124 | PNIQAVLLPKKTESHK_Acetyl_ | 1915.14 | High |
|  |  | PNIQAVLLPKKTESQK_Acetyl_TKSK | 2366.29 | High |
|  |  | AQGGVLPNIQAVLLPKKTESQK_Acetyl_TKSK | 3065.61 | High |
|  |  | AQGGVLPNIQAVLLPKKTESQK_Acetyl_TK | 2806.60 | High |
|  | K126 | AQGGVLPNIQAVLLPKKTESQKTK_Acetyl_ | 2806.60 | High |
|  | K128 | PNIQAVLLPKKTESQKTKSK_Acetyl_ | 2433.40 | High |
| H2b | K5 | PTK_Acetyl_SAPAPK | 980.54 | High |
|  |  | PTK_Acetyl_SAPAPK | 924.52 | High |
|  | K11 | PTKSAPAPK_Acetyl_ | 980.54 | High |
|  |  | PAPK_Acetyl_KGSKK | 1066.63 | High |
|  | K12 | K_Acetyl_GSKKAVTKAQK | 1441.84 | High |
|  |  | PAPKK_Acetyl_GSKK | 1066.63 | High |
|  | K15 | KGSK_Acetyl_KAVTKAQK | 1441.84 | High |
|  |  | GSK_Acetyl_KAVTKAQK | 1271.74 | High |
|  | K16 | KGSKK_Acetyl_AVTKAQK | 1441.84 | High |
|  |  | GSKK_Acetyl_AVTKAQK | 1271.74 | High |
|  |  | K_Acetyl_AVTKAQKK | 1127.68 | High |
|  |  | K_Acetyl_AVTKAQKKDGK | 1427.82 | High |
|  | K20 | AVTK_Acetyl_AQKK | 915.56 | High |
|  |  | TK_Acetyl_AQKK | 745.46 | High |
|  |  | GSKKAVTK_Acetyl_AQK | 1271.74 | High |
|  |  | KAVTK_Acetyl_AQKK | 1127.68 | High |
|  |  | KAVTK_Acetyl_AQKKDGK | 1427.82 | High |
|  |  | KGSKKAVTK_Acetyl_AQK | 1441.84 | High |
|  | K23 | KAVTKAQK_Acetyl_K | 1127.68 | High |
|  |  | KAVTKAQK_Acetyl_KDGK | 1427.82 | High |
|  | K27 | KDGK_Acetyl_KRK | 915.57 | High |
|  | K28 | KDGKK_Acetyl_RK | 915.58 | High |
|  | K34 | SRK_Acetyl_ESYSVYVYK | 1550.79 | High |
|  |  | K_Acetyl_ESYSVYVYKVLKQVHPDTGISSKAMGIMNSFVNDIFER | 4724.40 | High |
|  |  | K_Acetyl_ESYSVYVYK | 1307.65 | High |
|  | K43 | KESYSIYVYK_Acetyl_VLKQVHPDTGISSKAMGIMNSFVNDIFER | 4738.40 | High |
|  |  | KESYSVYVYK | 1307.66 | High |
|  |  | SYSVYVYK_Acetyl_VLKQVHPDTGISSKAMGIMNSFVNDIFER | 4481.28 | High |
|  | K46 | VLK_Acetyl_QVHPDTGISSKAMGIMNSFVNDIFER | 3349.69 | High |
|  |  | KESYSVYVYKVLK_Acetyl_QVHPDTGISSKAMGIMNSFVNDIFER | 4738.40 | High |
|  |  | VLK_Acetyl_QVHPDTGISSKAMGIMNSFVNDIFER | 3291.69 | High |
|  |  | K_Acetyl_QVHPDTGISSKAMGIMNSFVNDIFER | 3077.49 | High |
|  |  | VLK_Acetyl_QVHPDTGISSK | 1550.85 | High |
|  |  | SYSVYVYKVLK_Acetyl_QVHPDTGISSKAMGIMNSFVNDIFER | 4481.27 | High |
|  | K57 | ISSK_Acetyl_AMGIMNSFVNDIFER | 2201.04 | High |
|  |  | GISSK_Acetyl_AMGIMNSFVNDIFER | 2258.07 | High |
|  |  | SSK_Acetyl_AMGIMNSFVNDIFER | 2104.00 | High |
|  |  | SK_Acetyl_AMGIMNSFVNDIFER | 2032.93 | High |
|  |  | VLKQVHPDTGISSK_Acetyl_AMGIMNSFVNDIFER | 3291.69 | High |
|  |  | KESYSVYVYKVLKQVHPDTGISSK_Acetyl_AMGIMNSFVNDIFER | 4738.40 | High |
|  |  | SYSVYVYKVLKQVHPDTGISSK_Acetyl_AMGIMNSFVNDIFER | 4481.28 | High |
|  |  | QVHPDTGISSK_Acetyl_AMGIMNSFVNDIFER | 2949.39 | High |
|  |  | KQVHPDTGISSK_Acetyl_AMGIMNSFVNDIFER | 3095.44 | High |
|  | K108 | LPGELAK_Acetyl_HAVSEGTK | 1578.85 | High |
|  |  | STITSREIQTAVRLLLPGELAK_Acetyl_HAVSEGTK | 3303.89 | High |
|  |  | LLLPGELAK_Acetyl_HAVSEGTK | 1805.02 | High |
|  |  | PGELAK_Acetyl_HAVSEGTK | 1465.77 | High |
|  | K116 | PGELAKHAVSEGTK_Acetyl_ | 1507.78 | High |
| H3 | K9 | K_Acetyl_STGGKAPR | 985.55 | High |
|  | K14 | KSTGGK_Acetyl_APR | 971.57 | High |
|  |  | STGGK_Acetyl_APR | 815.44 | High |
|  | K18 | K_Acetyl_QLATKAAR | 1070.63 | High |
|  | K23 | KQLATK_Acetyl_AAR | 1028.63 | High |
|  |  | QLATK_Acetyl_AAR | 900.53 | High |
|  |  | KQLATK_Acetyl_AARKSAPSTGGVK | 2143.13 | High |
|  |  | ATK_Acetyl_AARKSAPSTGGVKKPHR | 2132.22 | High |
|  |  | KQLATK_Acetyl_AAR | 1070.63 | High |
|  | K27 | K_Acetyl_SAPSTGGVK | 973.53 | High |
|  |  | K_Acetyl_SAPSTGGVKKPHR | 1519.88 | High |
|  | K36 | SAPSTGGVK_Acetyl_KPHR | 1363.75 | High |
|  |  | KSAPSTGGVK_Acetyl_KPHR | 1505.86 | High |
|  |  | KQLATKAARKSAPSTGGVK_Acetyl_ | 2143.14 | High |
|  | K79 | EIAQDFK_Acetyl_TDLR | 1377.71 | High |
|  |  | K_Acetyl_TDLRFQSAAIGALQEASEAYLVGLFEDTNLCAIHAK | 4035.98 | High |
|  | K115 | FEDTNLCAIHAK_Acetyl_RVTIMPKDIQLAR | 2939.52 | High |
|  |  | YLVGLFEDTNLCAIHAK_Acetyl_ | 1948.98 | High |
|  |  | FQSAAIGALQEASEAYLVGLFEDTNLCAIHAK_Acetyl_RVTIMPKDIQ | 4674.45 | High |
|  | K122 | FQSAAIGALQEASEAYLVGLFEDTNLCAIHAKRVTIMPK_Acetyl_DIQ | 4674.45 | High |
| H4 | K5 | GK_Acetyl_GGKGLGKGGAKR | 1438.82 | High |
|  |  | GK_Acetyl_GGKGLGKGGAK | 1240.70 | High |
|  |  | GK_Acetyl_GGKGLGK | 885.52 | High |
|  | K8 | GGK_Acety_GLGKGGAKR | 1211.69 | High |
|  |  | GKGGK_Acetyl_GLGKGGAKR | 1396.81 | High |
|  |  | GKGGK_Acetyl_GLGKGGAK | 1198.69 | High |
|  |  | GGKG_Acetyl_LGKGGAK | 1013.58 | High |
|  |  | GKGGK_Acetyl_GLGK | 885.52 | High |
|  |  | GGK_Acety_GLGKGGAKRH | 1362.77 | High |
|  | K12 | KGGKGLGK_Acety_GGAKR | 1397.80 | High |
|  |  | GGKGLGK_Acety_GGAKR | 1211.69 | High |
|  |  | GGKGLGK_Acety_GGAKRH | 1362.77 | High |
|  |  | GGKGLGK_Acetyl_GGAK | 1013.58 | High |
|  |  | GKGGKGLGK_Acetyl_GGAK | 1198.69 | High |
|  |  | GLGK_Acetyl_GGAKR | 927.54 | High |
|  |  | GKGGKGLGK_Acetyl_GGAKR | 1396.81 | High |
|  | K16 | GGKGLGKGGAK_Acety_R | 1211.69 | High |
|  |  | GGKGLGKGGAK_Acety_RH | 1362.77 | High |
|  |  | GKGGKGLGKGGAK_Acetyl_R | 1396.81 | High |
|  |  | GLGKGGAK_Acetyl_R | 927.54 | High |
|  |  | KGGKGLGKGGAK_Acety_R | 1397.80 | High |
|  | K20 | K_Acety_VLRDNIQGITKPAIR | 1878.14 | High |
|  | K44 | K_Acetyl_RISGLIYEETRGVLKVFLENVIR | 2916.67 | High |
|  |  | VK_Acetyl_RISGLIYEETRGVLKVFLENVIR | 3203.87 | High |
|  | K59 | GVLK_Acetyl_VFLENVIR | 1428.87 | High |
|  |  | ISGLIYEETRGVLK_Acetyl_VFLENVIRDAVTYTEHAK | 3734.02 | High |
|  |  | VKRISGLIYEETRGVLK_Acetyl_VFLENVIR | 3203.87 | High |
|  | K77 | AK_Acetyl_RKTVTAMDVVYALKR | 2048.17 | High |
|  |  | AVTYTEHAK_Acetyl_RKTVTAMDVVYALKR | 2967.51 | High |
|  |  | DAVTYTEHAK_Acetyl_RKTVTAMDVVYALKR | 2950.51 | High |
|  |  | TYTEHAK_Acetyl_RKTVTAMDVVYALKR | 2623.42 | High |
|  |  | EHAK_Acetyl_RKTVTAMDVVYALKR | 2300.22 | High |
|  | K79 | TYTEHAKRK_Acetyl_TVTAMDVVYALKR | 2623.42 | High |
|  |  | AKRK_Acetyl_TVTAMDVVYALKR | 2048.17 | High |
|  |  | DAVTYTEHAKRK_Acetyl_TVTAMDVVYALKR | 2950.51 | High |
|  |  | AVTYTEHAKRK_Acetyl_TVTAMDVVYALKR | 2967.51 | High |
|  |  | AVTYTEHAKRK_Acetyl_TVTAMDVVYALK | 2665.37 | High |
|  |  | EHAKRK_Acetyl_TVTAMDVVYALKR | 2300.22 | High |
|  | K91 | IRDAVTYTEHAKRKTVTAMDVVYALK_Acetyl_ | 3035.66 | High |
|  |  | TVTAMDVVYALK_Acetyl_R | 1508.82 | High |

a: Displays the protonated monoisotopic mass of the peptides. It is the measured mass.

b: Confidence：Indicates a confidence level associated with the peptide sequence at the top level. The false discovery rate (FDR) is a statistical value that estimates the number of false positive identifications among all identifications found by a peptide identification search. Specifies the target false discovery rate for peptide matches of high confidence. Peptide matches that pass the filter associated with the strict FDR ( 0.01) indicates a high-confidence.
